# Jo-1 autoantigen-specific B cells are skewed towards distinct functional B cell subsets in anti-synthetase syndrome patients

**DOI:** 10.1186/s13075-020-02412-8

**Published:** 2021-01-19

**Authors:** Jennifer Young-Glazer, Alberto Cisneros, Erin M. Wilfong, Scott A. Smith, Leslie J. Crofford, Rachel H. Bonami

**Affiliations:** 1 Department of Medicine, Division of Rheumatology and Immunology, Vanderbilt University Medical Center, Medical Center North T3113, 1161 21st Avenue South, Nashville, TN 37232 USA; 2 Department of Medicine, Division of Allergy, Pulmonary, and Critical Care, Vanderbilt University Medical Center, Nashville, TN 37232 USA; 3 Department of Medicine, Division of Infectious Diseases, Vanderbilt University Medical Center, Nashville, TN 37232 USA; 4 Department of Pathology, Microbiology and Immunology, Vanderbilt University Medical Center, Nashville, TN 37232 USA

**Keywords:** Autoimmune diseases, B lymphocytes, Autoimmunity, Myositis

## Abstract

**Background:**

Anti-Jo-1 autoantibodies which recognize histidyl-tRNA synthetase identify patients with the rare rheumatologic disease, anti-histidyl-tRNA synthetase syndrome (Jo-1 ARS), a phenotypically distinct subset of idiopathic inflammatory myopathies (IIM). Jo-1-binding B cells (JBCs) are implicated in disease pathogenesis, yet they have not been studied directly. We therefore aimed to characterize JBCs to better understand how they expand and function in Jo-1 ARS.

**Methods:**

We enrolled 10 IIM patients diagnosed with Jo-1 ARS, 4 patients with non-Jo-1 IIM, and 8 age- and sex-matched healthy controls. We phenotypically characterized peripheral blood mononuclear cells (PBMCs) ex vivo using flow cytometry to define the B cell subsets in which JBCs reside. We further tested their ability to differentiate into antibody-secreting cells following stimulation in vitro.

**Results:**

The majority of JBCs were IgM^+^ (not class-switched). Compared to non-JBCs in the same donors, JBCs contained a higher percentage of autoimmune-prone CD21^lo^ cells and were increased in the CD21^lo^ IgM^+^ IgD^−^ CD27^+^ memory subset relative to healthy donor B cells. Whereas non-JBCs were present in the anergic B_ND_ B cell subset, JBCs were nearly absent from this compartment. JBCs were detected among plasmablasts in some donors, but a reduced frequency of JBCs differentiated into CD38^hi^24^−^ plasmablasts compared to non-JBCs present in the same wells following in vitro stimulation.

**Conclusions:**

JBCs are enriched for autoimmune-prone CD21^lo^ B cells, some of which exhibit a memory phenotype in the peripheral repertoire of Jo-1 ARS patients. JBCs undergo limited class switch and show reduced capacity to differentiate into antibody-secreting cells. This suggests complex B cell biology exists beyond class-switched cells that differentiate to secrete anti-Jo-1 autoantibody (i.e., what is captured through serum autoantibody studies). New Jo-1 ARS therapies should thus ideally target non-class-switched JBCs in addition to those that have undergone IgG class-switching to most effectively block cross-talk with autoreactive T cells.

**Supplementary Information:**

The online version contains supplementary material available at 10.1186/s13075-020-02412-8.

## Background

The idiopathic inflammatory myopathies (IIMs) are multi-system rheumatologic disease processes involving inflammation of skeletal muscles that affect 2.4–33.8 per 100,000 persons worldwide [1]. Classically, patients have been categorized as having dermatomyositis or polymyositis based on the presence or absence of cutaneous disease. However, the IIM phenotype can be more precisely characterized by distinct autoantibodies that correlate with clinical manifestations and comorbidities [2–4]. One subset of IIMs is known as “*a*nti t*R*NA *s*ynthetase syndrome” (ARS) due to autoantibodies that target specific tRNA synthetases. ARS manifestations frequently include interstitial lung disease, myositis, non-erosive arthritis, Raynaud’s phenomenon, and skin rashes [5]. The most common anti-tRNA autoantibody in ARS patients is anti-histidyl-tRNA synthetase (Jo-1) which defines Jo-1 ARS [4]. Jo-1 autoantibody levels correlate with increased Jo-1 ARS disease severity [6]. Despite the value of anti-Jo-1 humoral responses in guiding clinical management decisions, the characteristics and phenotype of Jo-1-binding B cells (JBCs), anti-Jo-1 autoantibody precursors, have not been defined.

Although histopathologic studies have implicated T cell-mediated tissue destruction in muscle damage [7], evidence suggests autoreactive B cells play an important role in this process. Serum measurements of B cell activating factor of the tumor necrosis factor family (BAFF) are increased in patients with IIM and correlate with anti-Jo-1 autoantibody titers and disease activity severity [6, 8]. B cell-targeting rituximab is commonly used to treat refractory ARS, and depletion of anti-Jo-1 autoantibodies correlates with clinical response [9, 10]. Clonal analysis of muscle-infiltrating B cells shows evidence of an antigen-driven immune response with somatic hypermutation, further providing evidence for B cell involvement in disease [11, 12].

The mechanism behind the immunologic response against the Jo-1 autoantigen and clinical manifestations of Jo-1 ARS is incompletely understood. Epitope heterogeneity suggests the mechanism is not simple molecular mimicry, but rather is directed against Jo-1 itself [13–15]. MHC class II haplotype and specific amino acids at particular positions within MHC class II molecules associate with Jo-1 autoantibody positivity [16, 17]; thus, Jo-1 autoantigen presentation to T cells contributes to Jo-1 ARS pathogenesis. Autoantibodies in patient sera demonstrate affinity maturation over time, further supporting an antigen-driven humoral response against Jo-1 [18]. The membrane-bound B cell receptor (BCR) is not automatically secreted as antibody; thus, Jo-1 autoantibodies may not mirror the full spectrum of JBCs competent to present Jo-1 autoantigen to T cells.

In this study, we established a cohort of Jo-1 ARS patients and developed methods to identify JBCs by high-throughput ELISA and flow cytometry. We phenotypically characterized JBCs and identified specific B cell subsets that serve as reservoirs for antigen-specific B cells. Given the identification of JBCs among both non-class-switched IgM and class-switched IgG populations, these data suggest the functional potential of JBCs as autoantigen-presenting cells should be considered independently of autoantibody production in monitoring expansion of autoreactive clones in disease, or alternatively their contraction as future experimental drug efficacy is assessed.

## Methods

### Patient selection and clinical information

Jo-1 ARS patients were selected from enrollees in the Myositis and Scleroderma Treatment and Investigative Center (MYSTIC) cohort at Vanderbilt University Medical Center as approved by the Vanderbilt Institutional Review Board. To capture all levels of disease severity, patients were recruited from outpatient rheumatology and pulmonology clinics, inpatient rheumatology and pulmonology consult services, and the intensive care unit. IIM patients were eligible for the study if they were 18 years or older and had been diagnosed with dermatomyositis or ARS by a rheumatologist or pulmonologist. Exclusions were pregnancy or HIV positivity. Twelve Jo-1 ARS patients were enrolled. One subject was excluded due to having subsequent negative Jo-1 testing from a commercial source, and one patient was excluded due to recent administration of rituximab. Of the remaining 10 subjects, five had active disease and five were noted to have stable disease. “Active disease” was defined by having immunosuppression increased during their outpatient visit or requiring hospitalization due to life-threatening disease. Subjects were defined as having “stable disease” if no medications were discussed during the encounter and current medications were either continued or de-escalated. Patients with active disease were typically earlier in their disease course with median enrollment 1.0 years after disease onset, while subjects with stable disease tended to have had the disease for a longer duration (median 13.8 years). Patients with active disease were typically being treated with high-dose steroids (80%), while stable subjects were using alternative steroid-sparing treatments including azathioprine, hydroxychloroquine, IVIG, and leflunomide (100%).

Four IIM subjects with alternative antibodies (anti-PL-7, anti-EJ with anti-Ro52, anti-Mi-2, and anti-TIF1-γ) were used as disease controls. Healthy participants recruited from the community were eligible if they were 18 years or older, had no personal history or first-degree relatives with autoimmune disease, and had no history of malignancy treated with immunomodulatory therapy. Healthy controls were age- and sex-matched with stable and active Jo-1 ARS cases. As shown in Table 1, the median age of subjects with active disease and stable disease was similar at 53.8 and 57.1 years, respectively, while the healthy subjects were slightly younger with a median age of 48.2. Our Jo-1 ARS cohort was predominately female (80%) and Caucasian (80%). Clinical, laboratory, medication, and radiologic electronic medical record data were collected. Data were managed using the REDCap software system (Vanderbilt).

**Table 1:** Study participant characteristics

	Healthy controls (*n* = 8)	Non-Jo-1 IIM* (*n* = 4)	Stable Jo-1+ disease** (*n* = 5)	Active Jo-1 disease*** (*n* = 5)
Age (years)	48.2 (42.1–52.0)	58.2 (50.9–63.2)	57.1 (43.4–57.4)	53.8 (46.2–56.1)
Sex (female)	5 (63)	3 (75)	5 (100)	3 (60)
Race				
Caucasian	6 (75)	4 (100)	5 (100)	3 (60)
African American	1 (13)	0	0	2 (40)
Other ethnicity	1 (13)	0	0	0
Disease duration (years)	–	2 (0.5–2.7)	13.8 (4.6–14.5)	1.0 (0.8–4.0)
Daily steroid dose ≥ 20 mg	–	3 (75)	0	4 (80)
Other immunosuppressant use	–	2 (50)^†^	5 (100)^‡^	1 (20)^§^

Data are presented as medians (interquartile ranges) and counts (percentages) for continuous and categorical data, respectively

*Anti-PL-7, anti-EJ with anti-Ro52, anti-Mi-2, and anti-TIF1-γ

**Stable Jo-1+ anti-synthetase syndrome defined as having no medication changes discussed, and current medications either continued or de-escalated at the time of blood draw

***Active Jo-1+ anti-synthetase syndrome defined as either immunosuppression increased during outpatient visit, or patient hospitalized with life-threatening disease at the time of blood draw

^†^IVIG (1), methotrexate (1)

^‡^Azathioprine (3), hydroxychloroquine (1), leflunomide (1)

^§^IVIG (1)

### Sample collection and processing

Peripheral blood mononuclear cells (PBMCs) were obtained by collecting whole blood into mononuclear cell preparation tubes with sodium heparin (BD) via peripheral venipuncture. Cells were washed twice in PBS and red blood cells were lysed using Ack Lysis Buffer (Gibco). Cells were again washed in PBS and counted. Cells were cooled in 10% dimethyl sulfoxide (DMSO) in fetal bovine serum (FBS) at a rate of − 1 °C/min until they reached a temperature of − 80 °C for 24–72 h. Cryopreserved PBMCs were stored in liquid nitrogen until the time of analysis and thawed with ~ 80% viability. To collect serum samples, peripheral venipuncture was performed and blood was collected into serum collection tubes with silica act clot activator (BD). After complete coagulation, tubes were spun and serum was frozen in aliquots at − 80 °C for future analysis.

### Recombinant protein cloning, expression, and purification

Online Supplemental Fig. S1 shows GST-Jo-1 fusion protein and GST-only (control) expression plasmids. *Homo sapiens* histidyl-tRNA synthetase (Jo-1), transcript variant 1 cDNA sequence (NM_002109.5) was purchased commercially (VB180802-1099xav, VectorBuilder) including an N-terminus GST-tag. The control vector was constructed similarly but only included the GST-tag sequence (VB181211-1124ant, VectorBuilder). Plasmids were transformed into BL21-CodonPlus (DE3)-RIL competent cells (Agilent). GST-Jo-1 and GST were expressed following induction with 0.1 mM IPTG for 16 h at 25 °C and affinity purified by Sepharose Glutathione beads (ThermoFisher). Online Supplemental Fig. S2 shows protein purity.

### ELISA

Three hundred eighty-four-well ELISA plates (Maxisorp) were coated with 1 μg/ml purified GST-Jo-1 or GST protein in 1X PBS overnight at 4 °C. Plates were blocked with 5% FBS in 1X PBS plus 0.5% Tween (1X PBS-T) at 25 °C and washed 5X with 1X PBS-T. Plates were incubated at RT with 1:1000 patient sera or stimulated PBMC culture supernatants diluted 1:2 in blocking buffer. Parallel wells were incubated with 100X soluble GST-Jo-1 competitor during sera binding to measure specific binding. Bound antibody was detected by anti-human IgG Fc-specific-HRP (Sigma), anti-human IgG-HRP, or anti-human IgM-HRP (Southern Biotech) secondary antibodies diluted in 5% FBS/1X PBS-T. Plates were washed, TMB Ultra ELISA substrate (Thermo Fisher) was added, and plates were read at O.D. 370 nm using a microplate reader (SpectraMax M3).

### B cell stimulation

Cryopreserved PBMCs were rapidly thawed, washed, and resuspended in ClonaCell-HY Medium A (StemCell). Cells were plated at a density of 0.033 × 10^6^ cells/ml in Medium A with 0.833 μg/ml of CpG + 0.133 μg/ml each of mouse-anti-human kappa and lambda antibodies (Southern Biotech). In addition, 0.033 × 10^6^ cells/ml viable gamma-irradiated NIH3T3 fibroblasts were added that had been genetically engineered to express cell-surface human CD40L and secrete human B cell activating factor (BAFF) and human IL-21. These stimuli drive B cells to secrete BCR as antibody to enable screening for antigen-specific B cells [19]. The mixture was then plated into 384-well flat bottom plates (Corning). Plates were incubated with 5% CO_2_ at 37 °C and screened for the presence of anti-Jo-1 antibody by ELISA on day 7, as described above.

### Flow cytometry

Cells were stained for flow cytometry analysis using the following reactive antibodies and reagents: CD38-AF700 (HIT2), CD27-APC (O323), CD21-PcP-Cy5.5 (Bu32), CD24-APC-Cy7 (ML5), CD10-PE (HI10a), IgM-Pacific Blue (MHM-88), CD5-BV785 (UCHT2), CD3-BV510 (OKT3), CD14-BV510 (M5E2), CD16-BV510 (3G8), CD19-BUV395 (SJ25C1), IgG-PE-Cy7 (M131G05), and IgD-FITC (Ia6-2) (BioLegend, BD Biosciences, eBioscience, or Tonbo Biosciences). Jo-1 binding cells were identified through staining with GST-Jo-1 followed by detection with anti-GST-Texas Red (Abcam). Samples were acquired using a LSR II flow cytometer (BD Biosciences) and data were analyzed using FlowJo software (Tree Star). Flow cytometry samples identifying less than 20 JBC events were excluded from downstream analysis of B cell subset composition. Non-JBC subset analysis was still performed for these donors.

### Statistical analysis

All statistical analysis was performed using GraphPad Prism software version 8.3.1 (GraphPad Software). The resulting data are expressed as the mean frequency of the parent population ± standard deviation. Differences across B lymphocyte populations for a given phenotype were tested using a Mann-Whitney *U* test.

## Results

### High-throughput screening detects JBCs in the peripheral blood of Jo-1 ARS patients with both inactive and active disease

**Fig. 1:**
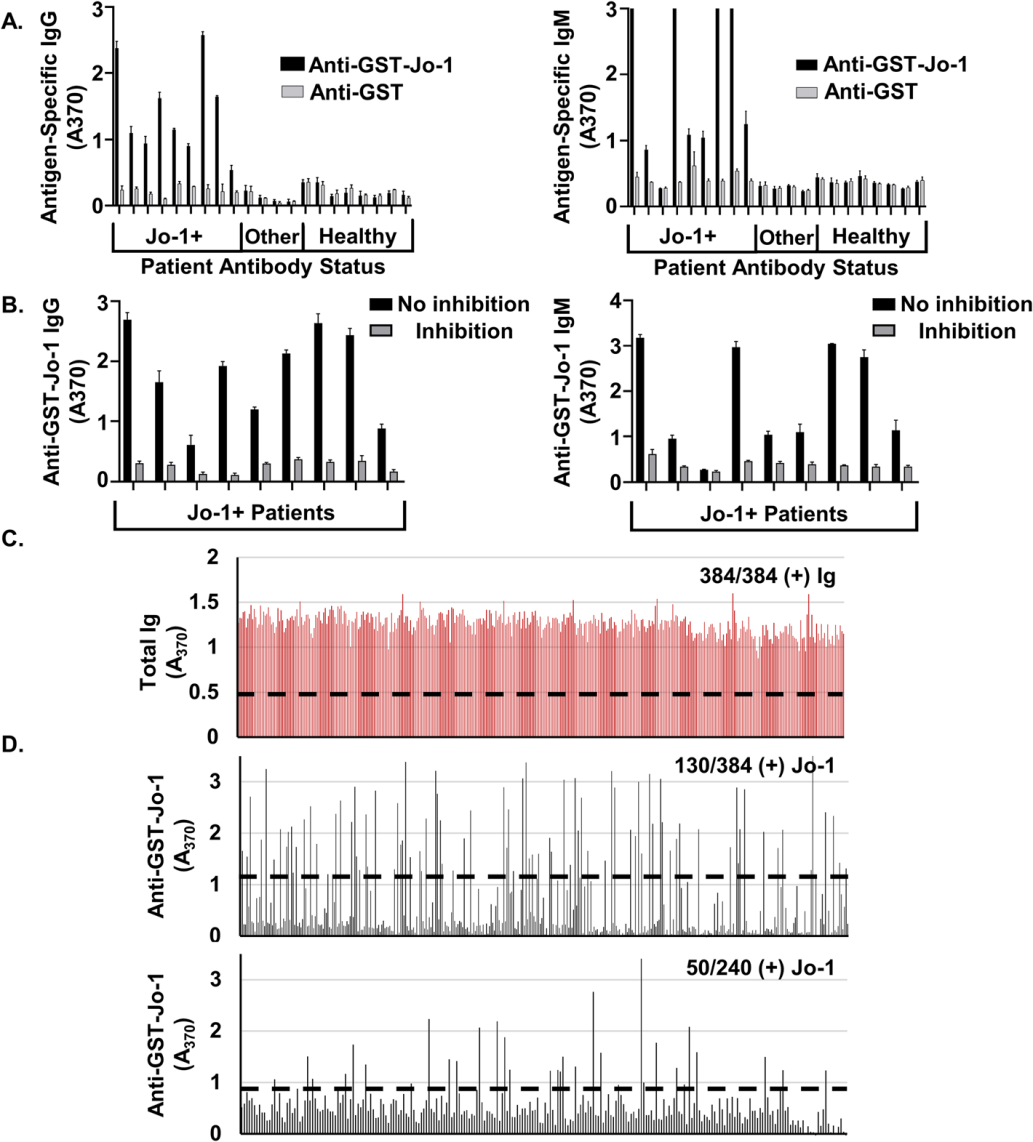
Jo-1-specific antibody is detected in sera and stimulated PBMC cultures from subjects with Jo-1 ARS. Serum and PBMCs were collected from individuals with clinical laboratory-confirmed cases of Jo-1 ARS disease (*n* = 9), IIM patients with “Other Ab” (anti-PL-7, anti-EJ with anti-Ro52, anti-Mi-2, and anti-TIF1- γ), and healthy subjects (*n* = 8). **a** ELISA was used to measure serum IgG (left) or IgM (right) antibody bound to purified GST-Jo-1 fusion protein (black) and purified GST protein (gray). **b** ELISA was used to measure serum IgG (left) or IgM (right) antibody bound to GST-Jo-1 in the absence (black) or presence of excess soluble GST-Jo-1 competitor (gray) to detect Jo-1-specific autoantibody. **c**, **d** PBMCs collected from *n* = 5 Jo-1+ subjects were separated into individual wells and polyclonally stimulated as in the “Methods” section to drive B cell differentiation into antibody-secreting cells. The fraction of positive wells is shown secreting **c** total antibody or **d** anti-Jo-1. **d** Results from two representative patients are shown. Dashed line defines positive wells with a mean fluorescence that is 3 standard deviations above the mean O.D. of all wells

Our ELISA assay detects serum anti-Jo-1 IgG and distinguishes Jo-1+ disease from healthy control donors (*A*_370_ mean difference of 1.22 ± 0.25, *p* < 0.0001, Fig. 1a). One subject did not have sera collected and was thus excluded. To confirm specificity of our ELISA assay, 4 IIM subjects with non-Jo-1 disease were also tested and were negative for anti-Jo-1 autoantibody (Fig. 1a). A GST-only control produced minimal signal compared to GST-Jo-1 (*A*_370_ mean difference of 1.20 ± 0.02, *p* < 0.0001), indicating signal was specific to the Jo-1 portion of the fusion protein. Fluorescence was reduced by a mean of 84% when 200-fold excess competitor Jo-1-GST protein was co-incubated with patient sera, further demonstrating the specificity of the assay to detect anti-Jo-1 IgG (Fig. 1b). Inhibitable, Jo-1-specific IgM was also found in Jo-1 ARS patients, but not in IIM subjects with non-Jo-1 disease (Fig. 1a, b). These data show that Jo-1 autoantibody is produced by class-switched (IgG^+^), as well as non-class-switched (IgM^+^) B cells in Jo-1 ARS patients.

To identify JBCs in the peripheral blood of Jo-1 ARS patients, we used a high-throughput method capable of capturing antigen-specific B cells as rare as 1 in 20 million cells [19]. This stimulation protocol drives B cells to secrete the BCR as antibody so that wells containing antigen-specific B cells can be identified using ELISA screening. The majority of wells secreted immunoglobulin (99.9%, Fig. 1c), including both IgG and IgM (99.9%, Fig. 1c and Fig. S3). Figure 1d shows identification of wells that contained JBCs using PBMCs isolated from two representative Jo-1 ARS patients. On average, 20.33% of stimulated wells produced anti-Jo-1 autoantibodies (data not shown). Therefore, JBCs are present in the peripheral blood repertoire of these subjects.

### The majority of JBCs are not class-switched but are found in both IgM^+^ and IgG^+^ B cell subsets in Jo-1 ARS patients with both stable and active disease

**Fig. 2:**
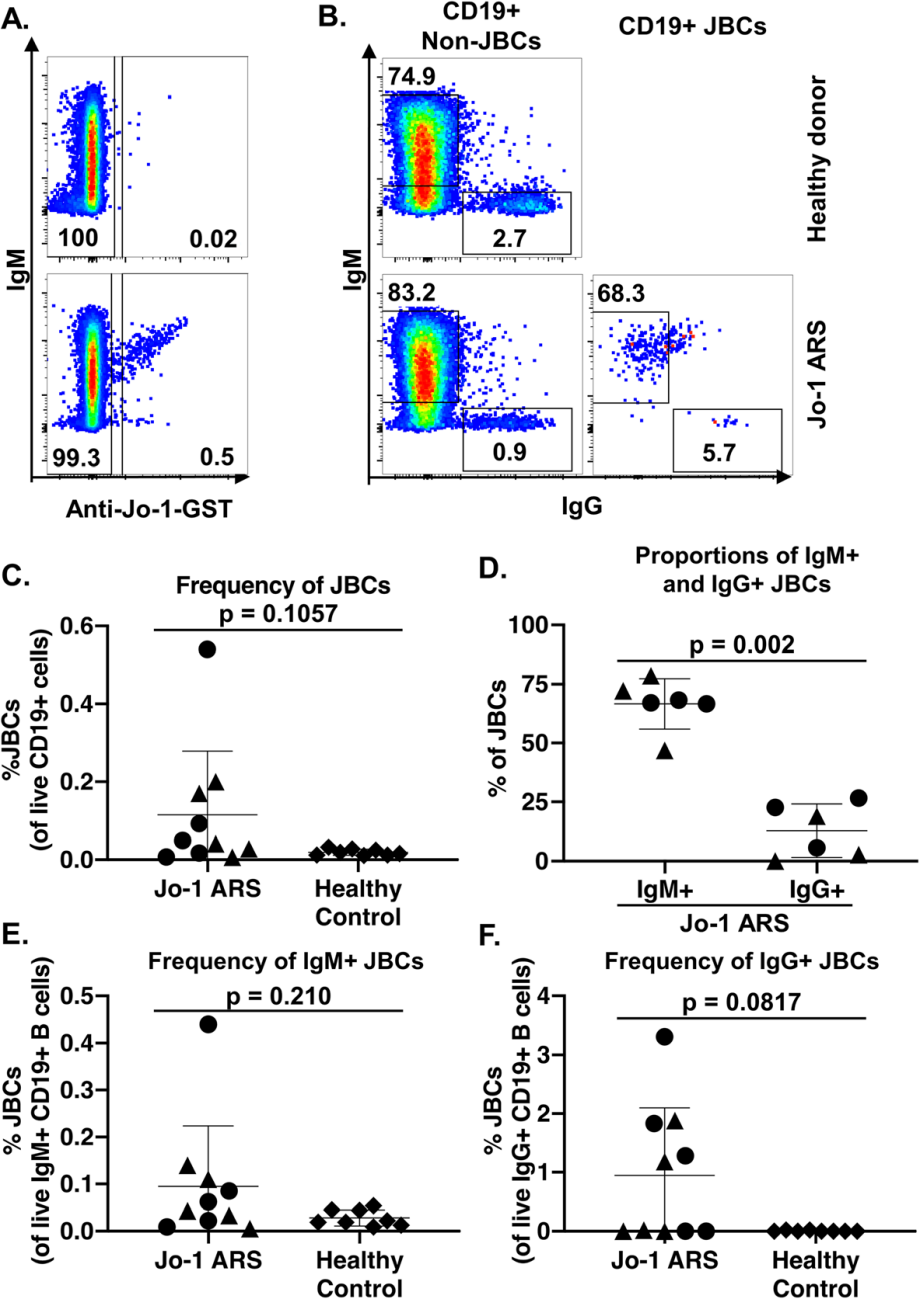
JBCs are detected among IgM^+^ and IgG^+^ B cells, with the majority being non-class-switched. PBMCs from healthy controls (top) or Jo-1 ARS (bottom) patients were stained and analyzed using flow cytometry. **a** Live, CD19^+^ CD5^−^ CD14^−^ CD16^−^ cells were gated and anti-GST secondary antibody discriminated between Jo-1/GST-binding (Jo-1^+^) and non-JBCs (Jo-1^−^) B cells. **b** Cells were further gated on IgM versus IgG expression by non-JBC and JBC. Representative plots from healthy control and Jo-1 ARS donors are shown. **c**–**f** Flow cytometry identifies the indicated populations among *n* = 5 stable Jo-1 ARS (triangles), *n* = 5 active Jo-1 ARS (circles), and *n* = 8 healthy controls (diamonds). **c** JBCs were identified as in panel **a** and their frequency of total B cells was determined. **d** The IgM^+^ or IgG^+^ cell frequency (gated as in panel **b**) of JBCs (gated as in panel **a**) is shown for Jo-1 ARS patients that had > 20 JBC events (*n* = 6). **e** The frequency of JBCs within IgM^+^ (**e**) or IgG^+^ (**f**) B cells is shown. Individual donors are plotted, and bars represent the mean ± SD. *p* values were determined using the Mann-Whitney *U* test, and are indicated on each panel

We next sought to measure the frequency of circulating JBCs in Jo-1 ARS patients and phenotypically characterize these cells ex vivo. In order to account for disease progression as a potential driving force behind cell phenotypic variation, we used flow cytometry to analyze PBMCs from Jo-1 ARS donors whose disease was progressing (active disease, *n* = 5), donors whose disease is not progressing (stable disease, *n* = 5), and healthy controls (*n* = 8). These samples were matched according to age and sex. JBCs were readily identified in a subset of Jo-1 ARS patients with active disease and stable disease, but not healthy controls (Fig. 2a, c). The majority of JBCs were IgM^+^ (Fig. 2a, d; mean ± SD, 66.6 ± 10.7% IgM^+^ vs 12.8 ± 11.3% IgG^+^; *p* = 0.0022). We further measured the frequency of JBCs within IgM^+^ and IgG^+^ B cells. IgM^+^ and IgG class-switched JBCs were observed in both stable and active disease patients (Fig. 2e, f).

### JBCs are biased away from the anergic B_ND_ subset but towards CD21^lo^ B cells

**Fig. 3:**
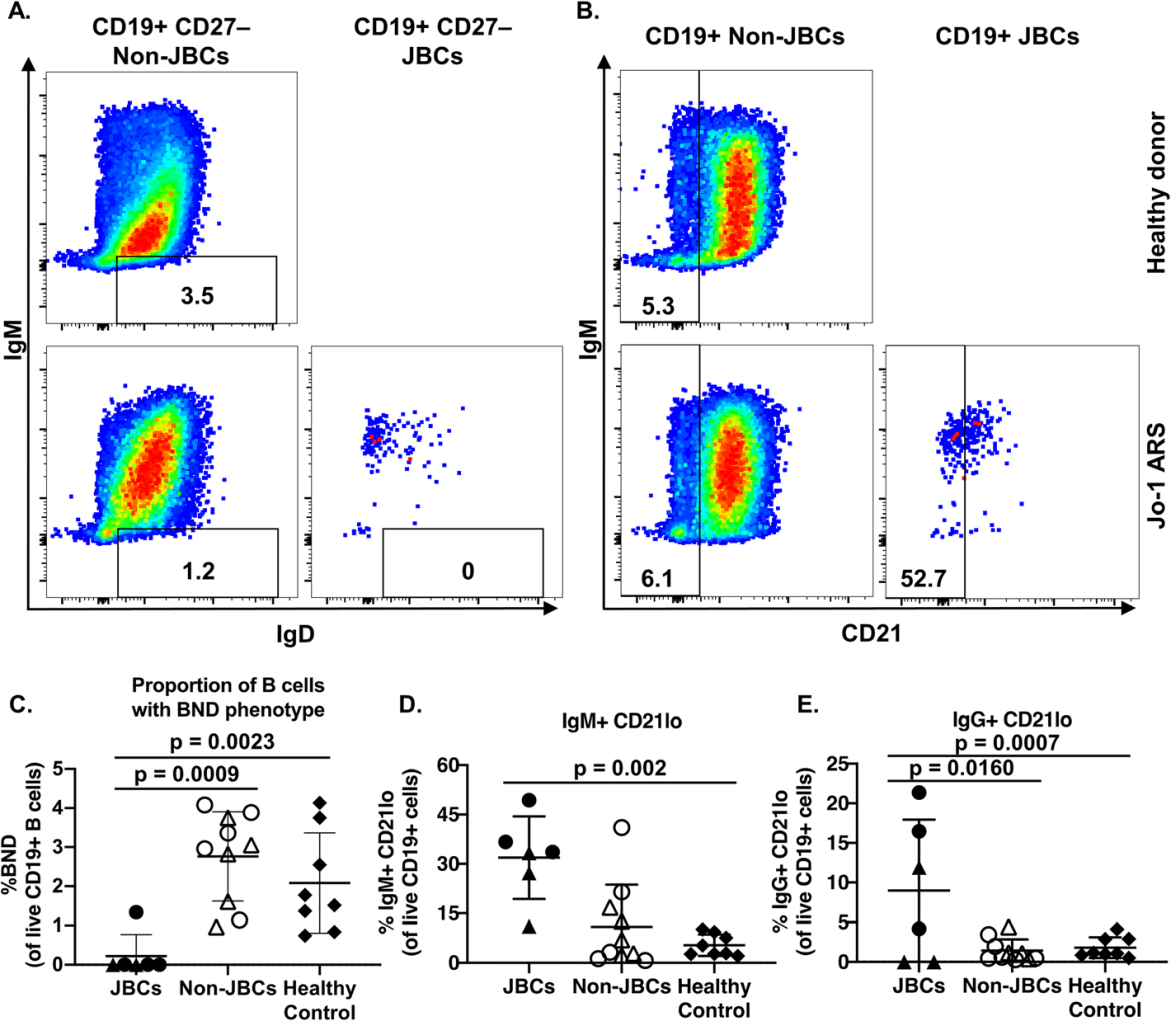
JBCs are enriched among CD21^lo^, but not B_ND_ B cell subsets. PBMCs from healthy controls or Jo-1 ARS patients were stained and analyzed using flow cytometry. Live B cells were identified as in Fig. 2 and **a** B_ND_ (IgM^lo^ IgD^−^ CD27^−^) or **b** CD21^lo^ B cells were identified; representative plots are shown for healthy controls (top) or Jo-1 ARS patients (bottom). **c**–**e** *n* = 5 stable Jo-1 ARS (triangles), *n* = 5 active Jo-1 ARS (circles), and *n* = 8 healthy controls (diamonds). Only those Jo-1 ARS patients that had > 20 JBC events (*n*=6) were included for JBC sub-analysis. **c** The frequency of B_ND_ B cells among JBCs and non-JBCs in Jo-1 ARS patients (identified as in Fig. 2) or from healthy donors is shown. **d**, **e** CD21^lo^ B cells were further gated on **d** IgM^+^ or **e** IgG^+^ cells and the frequencies of the indicated subsets are shown. Individual donors are plotted, and bars represent the mean ± SD. *p* values were determined using the Mann-Whitney *U* test, and significant values are indicated on each panel

Peripheral immune tolerance can render autoreactive B cells anergic in both mice and humans [20–23]. B_ND_ B cells (IgD^+^ IgM^lo^ CD27^−^, Table 2) were shown previously to be functionally anergic [24, 25]. We therefore investigated whether JBCs were enriched among this subset. JBCs were skewed away from the B_ND_ B cell subset compared to non-JBCs or healthy control B cells (Fig. 3a, c; 0.22 ± 0.55% versus 2.76 ± 1.14%; *p* = 0.0009 (non-JBCs) and 2.083 ± 1.28%; *p* = 0.0023 (healthy)).

**Table 2:** B cell phenotype definitions

B cell type	Population phenotype
**JBCs**	CD19^+^ Jo-1-GST^+^
**Non-JBCs**	CD19^+^ Jo-1-GST^−^
**B**_**ND**_	CD19^+^ CD27^−^ IgD^+^ IgM^lo/−^
**CD21**^**lo**^	CD19^+^ CD21^lo/−^
**CD21**^**lo**^ **CD27**^**+**^ **memory**	CD21^lo^ CD27^+^ IgD^−^
**Unswitched memory**	CD19^+^ CD27^+^ IgD^+^ IgM^+^
**Switched memory**	CD19^+^ CD27^+^ IgD^−^ IgM^−^ IgG^+^
**IgM memory**	CD19^+^ CD27^+^ IgD^−^ IgM^+^
**Atypical memory**	CD19^+^ CD24^−^ CD27^−^ CD38^−^ IgD^−^
**Transitional**	CD19^+^ CD10^+^ CD24^hi^ CD27^−^ CD38^+^ IgD^+^
**Plasmablasts**	CD19^+^ CD24^−^ CD38^hi^

All populations were additionally gated on live, singlet lymphocytes

CD21^lo^ B cells are a heterogeneous subset that have been reported to have functional properties of anergic, recently activated, or memory B cells [20, 26–29]. Previous studies showed increased IgM^+^ CD21^lo^ B cells in rheumatoid arthritis (RA) and Sjögren’s syndrome (SjS) patients [20, 30]. We therefore investigated whether an increased frequency of CD21^lo^ B cells was present among JBCs and compared Jo-1 ARS patients with healthy controls. Autoreactive-prone CD21^lo^ B cells are typically IgM^+^; however, IgG^+^ CD21^lo^ B cells have also been identified [20, 27, 30]. JBCs and non-JBCs were identified in the same donor and the frequencies of B cell phenotypic subsets were compared (Fig. 3b). We found that JBCs contain a higher proportion of CD21^lo^ B cells than non-JBCs (Fig. 3b, d, e; IgM, *p* < 0.01 and IgG, *p* < 0.05).

### JBCs are skewed towards CD21^lo^ non-switched CD27^+^ memory B cells

**Fig. 4:**
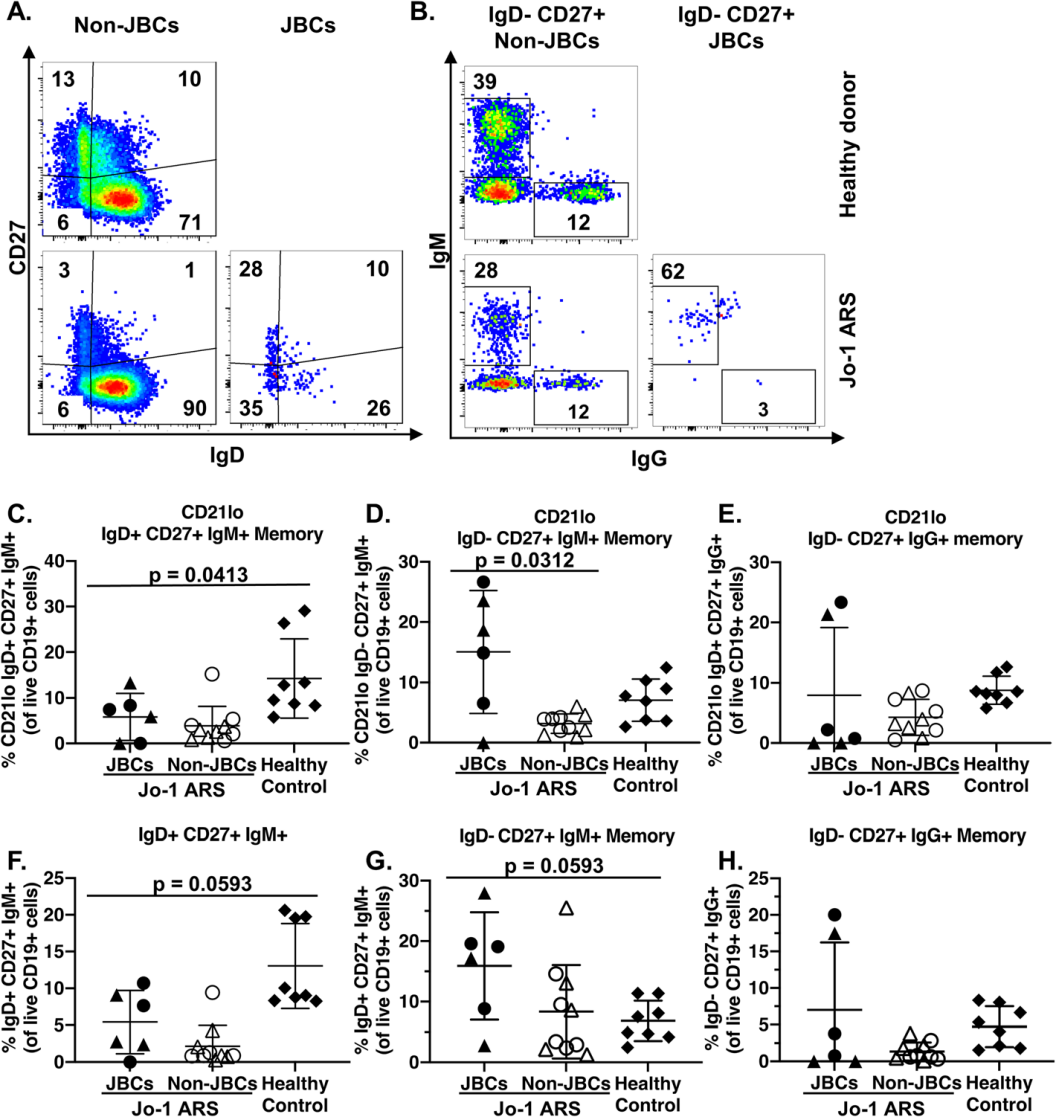
JBCs are enriched among CD21^lo^ IgM^+^ memory cells. PBMCs from healthy controls (top) or Jo-1 ARS (bottom) patients were stained and analyzed based on IgD, IgM, IgG, and CD27 expression using flow cytometry. Live JBCs and non-JBCs (Jo-1 ARS) or total B cells (healthy donors) were identified as in Fig. 2. **a**, **b** Representative plots are shown for healthy control (top) or Jo-1 ARS (bottom) donors for the indicated markers. IgM and IgG expression was examined among CD27^+^ IgD^−^ cells gated as in panel **a**. **c**–**h** The indicated phenotypic subsets were examined in *n* = 5 stable Jo-1 ARS (triangles), *n* = 5 active Jo-1 ARS (circles), and *n* = 8 healthy controls (diamonds). Only those Jo-1 ARS patients that had > 20 JBC events were included for JBC sub-analysis of either CD21^lo^ (**c**–**e**) or all cells (**f**–**h**) for **c**, **f** IgD^+^ CD27^+^ IgM^+^, **d**, **g** IgD^−^ CD27^+^ IgM^+^, and **e**, **h** IgD^−^ CD27^+^ IgG^+^ subsets. Individual donors are plotted, and bars represent the mean ± SD. *p* values were determined using the Mann-Whitney *U* test, and significant values are indicated on each panel

Memory B cells have complex phenotypes. We investigated memory markers among CD21^lo^ B cells as shown in Fig. 4a and b and previously described [31]. Unswitched CD21^lo^ memory (CD21^lo^ IgM^+^ IgD^+^ CD27^+^) B cells were decreased in JBCs when compared to healthy controls (Fig. 4c, *p* < 0.05), though no difference was found between JBCs and non-JBCs (*p* = 0.54). CD21^lo^ IgM only memory cells (CD21^lo^ IgM+ IgD− CD27+) were increased in JBCs when relative to non-JBCs (Fig. 4d, *p* = 0.0312), but not when compared to healthy control B cells (*p* = 0.18). No difference was observed in the frequency of IgG-switched CD21^lo^ memory between JBCs and non-JBCs or healthy cells (Fig. 4e, *p* = 0.54 (non-JBCs) and *p* = 0.32 (healthy controls)).

No difference was observed between JBCs and non-JBCs or healthy donors for unswitched (IgM^+^ IgD^+^) memory (Fig. 4f, *p* = 0.15 (non-JBCs) and *p* = 0.06 (healthy controls)), IgM^+^ IgD^−^ CD27^+^ (Fig. 4g, *p* = 0.06 (non-JBCs) and *p* = 0.09 (healthy controls)), IgD^−^ CD27^+^ IgG^+^ (Fig. 4h, *p* = 0.6901 (non-JBCs) and *p* = 0.55 (healthy)), or IgD^-^ CD27^-^ CD24^-^ CD38^-^ (Figure S4c) subsets. JBCs are therefore enriched for CD21^lo^ IgM^+^ memory B cells, a phenomenon that depends on autoantigen specificity of the B cell, rather than general differences in donor skewing towards these B cell subsets.

### JBCs do not show transitional B cell enrichment

**Fig. 5:**
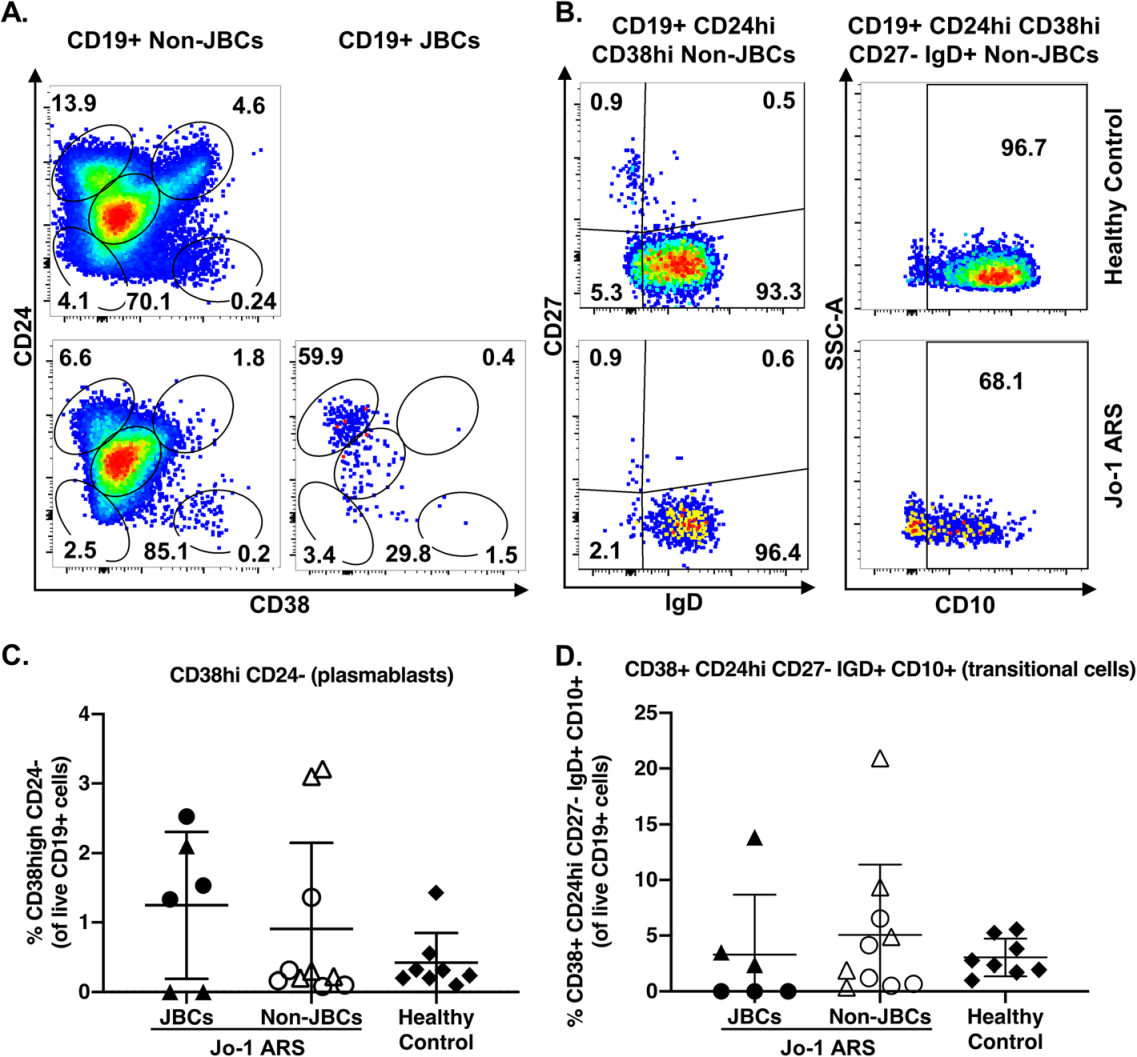
JBCs are not increased among transitional B cells or plasmablasts. PBMCs from healthy controls (top) or Jo-1 ARS (bottom) patients were stained and transitional B cells (CD24^hi^ CD38^hi^ CD27^−^ IgD^+^ CD10^+^) or plasmablasts (CD24^−^ CD38^high^) were identified using flow cytometry. **a**, **b** Representative plots are shown for healthy control (top) or Jo-1 ARS (bottom) donors for the indicated markers. **b** CD24^hi^ CD38^hi^ cells as gated in panel **a** are further gated on IgD/CD27 (left) and the resulting IgD^+^ CD27^−^ cells are gated on CD10 (right). **c**, **d** The indicated phenotypic subsets were examined in *n* = 5 stable Jo-1 ARS (triangles), *n* = 5 active Jo-1 ARS (circles), and *n* = 8 healthy controls (diamonds). Only those Jo-1 ARS patients that had > 20 JBC events were included for JBC sub-analysis of **c** plasmablasts or **d** transitional B cells. Individual donors are plotted, and bars represent the mean ± SD. *p* values were determined using the Mann-Whitney *U* test, and significant values are indicated on each panel

Fifty to 75% of transitional B cells (Table 2) are autoreactive, which are culled by immune checkpoints in healthy individuals [32]. These checkpoints function less efficiently in patients with autoimmunity, resulting in increased transitional B cells in the peripheral blood of patients with other rheumatic diseases [33–36]. To test whether a higher proportion of JBCs are transitional B cells in Jo-1 ARS, we measured this subset among JBCs and non-JBCs from the same Jo-1 ARS donors as well as healthy controls. While not significant, JBCs from Jo-1 ARS subjects trended towards having a higher frequency of transitional B cells relative to healthy controls (Fig. 5a, b, d, 3.3 ± 5.4% versus 3.0 ± 1.7%; *p* = 0.41).

### JBCs do not show plasmablast enrichment compared to non-JBCs or healthy donors

B cells differentiate into short-lived plasmablasts during acute immune responses; thus, plasmablasts can contain a relatively high proportion of antigen-specific B cells [37, 38]. Given the importance of Jo-1 autoantibody in predicting clinical phenotype [4], we tested whether JBCs showed an increased frequency of plasmablasts (CD38^hi^CD24^−^). Contrary to our expectations, the frequency of CD38^hi^CD24^−^ plasmablasts was not increased among JBCs relative to non-JBCs from the same donors, or healthy controls (Fig. 5a, c, JBCs versus non-JBCs, *p* = 0.93 and healthy controls *p* = 0.39). JBCs were found among IgM and IgG plasmablasts (not shown).

### A lower frequency of JBCs acquire a plasmablast phenotype following stimulation in vitro

**Fig. 6:**
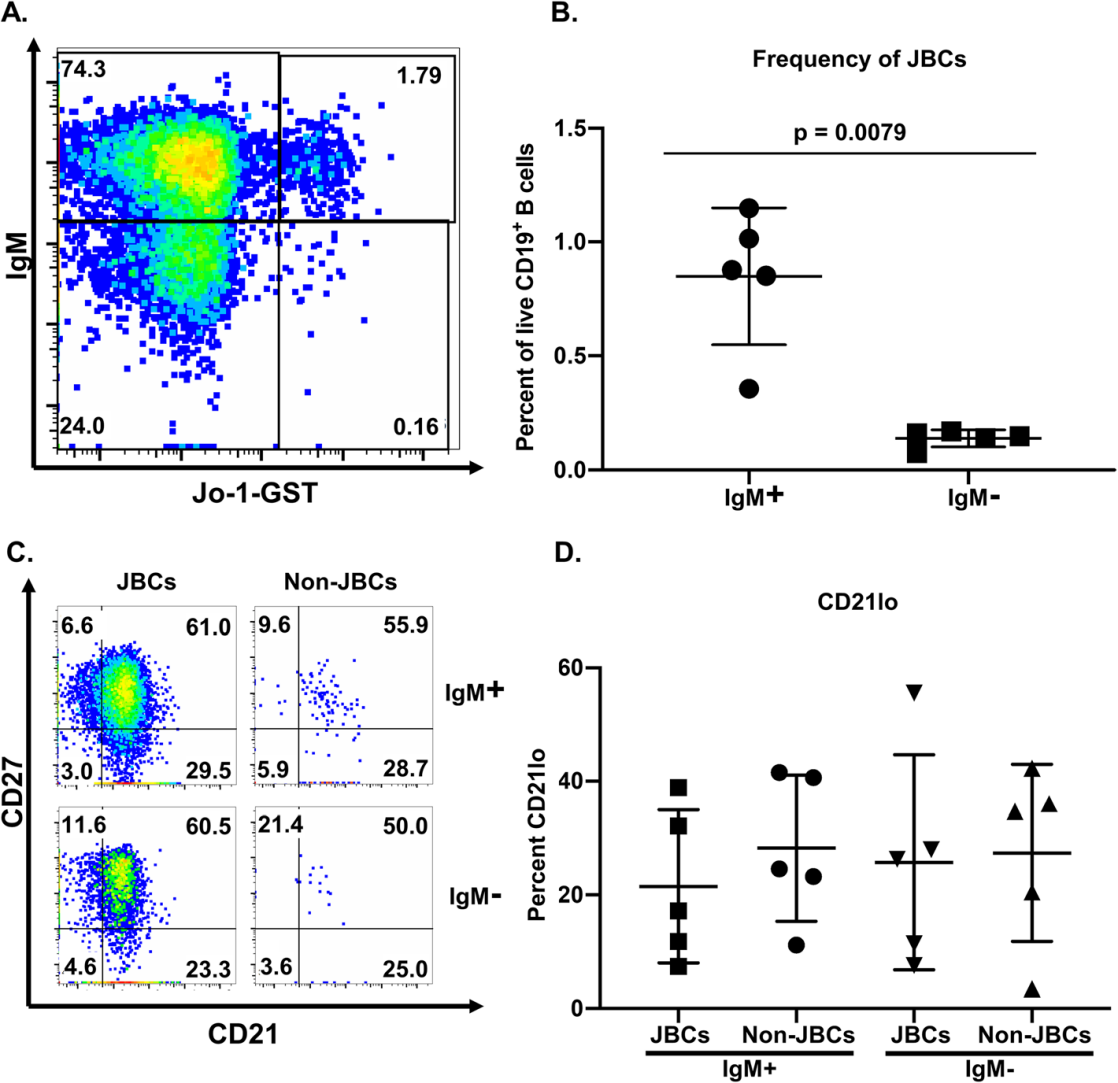
JBCs are identified following stimulation in vitro but are predominantly IgM^+^ (non-class-switched). PBMCs from Jo-1 ARS patients with active disease were stimulated as described in the “Methods” section. Cultured cells from 40 to 60 wells per patient were harvested on day 6 and stained for flow cytometry analysis. **a** Representative pseudocolor plots describing the gating strategy are shown for the selection of live, CD19^+^ IgM^+/−^ Jo-1 binding populations. The frequencies for each of these plots are indicated. **b** Cells were gated on live, CD19^+^ JBCs and the mean frequency of IgM^+^ and IgM^−^ cells was calculated per donor; individual donors are plotted. **c** Pseudocolor plot depicting the CD21^lo^ B cells in JBCs and non-JBCs. **d** The mean frequency of CD21^lo^ B cells within the indicated populations was calculated for per donor and individual donors are plotted. Statistical significance between populations as calculated using the Mann-Whitney *U* test is indicated. Bars indicate the mean ± SD

To test the functional capacity of JBCs, we stimulated PBMCs isolated from Jo-1 ARS patients with active disease as in Fig. 1 to drive their differentiation into antibody-secreting cells. After 6 days of stimulation, we used flow cytometry to determine the phenotype of JBCs versus non-JBCs from the same donors. Both JBCs and non-JBCs expanded following polyclonal BCR stimulation (not shown). We identified JBCs and non-JBCs within wells derived from individual patients and compared the resulting phenotypic subsets across patients. Consistent with our ex vivo findings (Fig. 2), the majority of JBCs were IgM^+^ following stimulation (*p* = 0.0079, Fig. 6a, b).

**Fig. 7:**
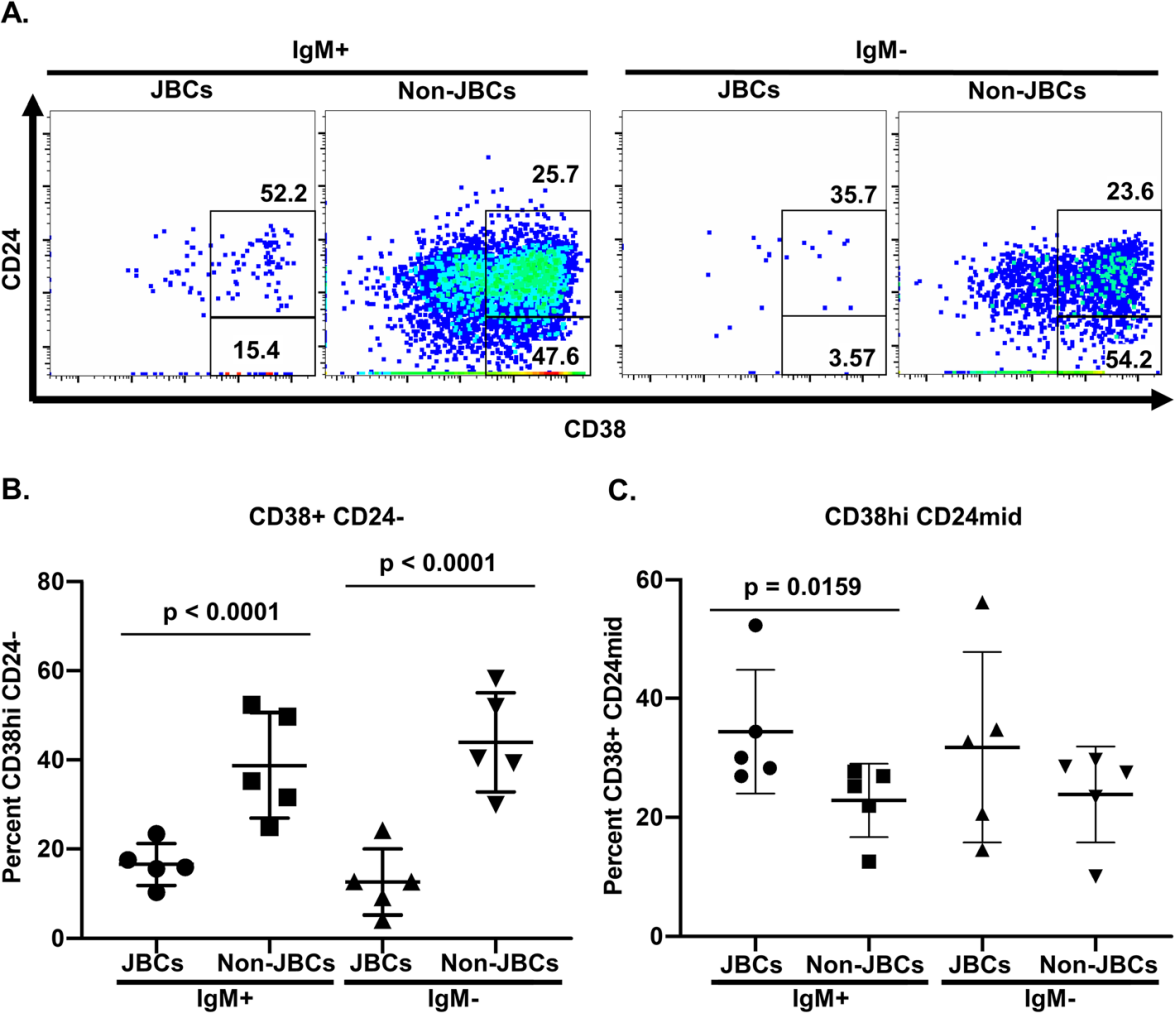
A lower frequency of JBCs acquire a plasmablast phenotype following stimulation in vitro compared to non-JBCs from the same Jo-1 ARS patients. PBMCs from *n* = 5 Jo-1 ARS patients with active disease were polyclonally stimulated as in the “Methods” section. Cultured cells from n=40-60 wells per patient were harvested on day 6 and stained for flow cytometry analysis. **a** CD24 and CD38 expression is shown in representative flow plots derived from a single well of stimulated PBMCs. Comparison of mean frequencies of **b** CD38^hi^ CD24^−^ B lymphocytes or **c** CD38^hi^ CD24^mid^ B lymphocytes between JBC and non-JBC populations. Significant differences between IgM^+^ JBC and IgM^+^ non-JBC or IgM^−^ JBC and IgM^−^ non-JBC populations as calculated using Mann-Whitney *U* test are indicated. The data are represented as the mean for each population within a given stimulation, and the bars indicate the mean ± SD

The proportion of CD21^lo^ B cells did not differ between JBC and non-JBC populations (Fig. 6c, d, IgM^+^ JBCs vs. IgM^+^ non-JBCs, *p =* 0.42; IgM^−^ JBCs vs. IgM^−^ non-JBCs, *p* = 0.84). In vitro stimulation reduced CD21 expression across all B cells, compared to CD21 levels on B cells ex vivo (not shown); thus, CD21^lo^ B cell differences could be masked in this setting. A comparison of relative frequencies for CD19^+^ CD38^hi^ CD24^−^ cells revealed a smaller proportion of cells with a plasmablast phenotype among JBCs relative to non-JBCs (Fig. 7a, b; IgM^+^ JBCs vs IgM^+^ non-JBCs, *p * < 0.0001), consistent with ex vivo findings (Fig. 55). This was also observed among IgM^−^ (class-switched) cells (IgM^−^ JBCs vs IgM^−^ non-JBCs, *p*   <0.0001). These stimulated populations contained a substantial proportion of CD38^hi^ cells with intermediate CD24 expression, designated CD38^hi^ CD24^mid^. A significantly larger proportion of IgM^+^ JBCs expressed this phenotype when compared to IgM^+^ non-JBCs (Fig. 7a, c; IgM^+^ JBCs vs IgM^+^ non-JBCs, *p =* 0.015), indicating that while JBCs show less downregulation of CD24, most upregulate CD38 following in vitro stimulation (Figs. 6 and 7).

## Discussion

In this study, we developed methods to identify JBCs and show they are present in the peripheral blood of patients with Jo-1 ARS among both IgM^+^ and IgG^+^ B cells. We find JBCs are enriched for CD21^lo^ B cells, a subset that is globally elevated among total B cells in other rheumatic diseases [20, 39]. Our samples were collected from patients with differences in disease severity, including patients requiring hospitalization and intensive care that would not be captured by outpatient enrollment. The ability to compare between JBCs and the remaining non-JBC repertoire within the same patient reduces the influence of confounding variables such as sex, age, ethnicity, individual immune variation, and treatment effects. Targeting autoantigen-specific B cells was successfully applied to mice to prevent autoimmune disease and is an attractive strategy to treat human autoimmunity [40, 41]. JBCs are thus putative therapeutic targets in Jo-1 ARS. Overall, these data advance the understanding of antigen-specific B cell biology in Jo-1 ARS by extending their potential autoimmune contributions beyond IgG class-switching and antibody production.

We detect IgM^+^ JBCs in peripheral blood, which is consistent with ARS/IIM patient muscle biopsy findings that suggest IgG class switch and terminal differentiation of JBCs occurs at the site(s) of attack [11, 12, 42]. The majority of JBCs identified in peripheral blood had not undergone class switch. Tetanus toxoid responses in hyperimmunized individuals also skew towards IgM in the peripheral blood; tetanus toxoid-specific B cell enrichment is primarily observed among IgG^+^ B cells proximal to vaccination and does not persist [43]. We identified 5/10 Jo-1 ARS patients that had clear IgG^+^ JBCs in peripheral blood, ranging from 1 to 3% of total IgG^+^ B cells; this population was found in both stable and active disease patients. This suggests class-switching is likely ongoing in Jo-1 ARS disease and is not restricted to a rare immune tolerance breach event resulting in a clonal burst of IgG-switched JBCs. This finding suggests that IgG class-switching of JBCs is not restricted to tissue and/or that they exit tissues to recirculate after undergoing IgG class switch.

Defective tolerance checkpoints allow increased escape of autoreactive B cells from the bone marrow into the transitional compartment in the periphery in multiple autoimmune diseases, including RA, SjS, type 1 diabetes, and systemic lupus erythematosus (SLE), thereby increasing the probability of developing autoantibodies [33–36]. In juvenile dermatomyositis, immature transitional B cells are expanded in patients with active disease and have an inflammatory phenotype with high interferon alpha and TLR7-pathway signals [44]. Whereas one patient with severe disease shows an elevated percentage of transitional B cells among JBCs, a larger Jo-1 ARS patient cohort will be required to ascertain the extent to which the developmental block observed in other autoimmune diseases impacts JBCs in Jo-1 ARS.

Autoantigen-binding B cells are absent from the anergic (B_ND_) B cell compartment in pre-diabetic and new-onset type 1 diabetes subjects and autoimmune thyroid disease patients, suggesting escape from this tolerant state is a harbinger of fulminant autoimmunity [45, 46]. Furthermore, loss of B_ND_ B cells in type 1 diabetes donors is associated with high-risk MHC class II alleles, suggesting interaction with T cells is involved in this process [46] and that B_ND_ B cells may regain functionality to present antigen to T cells. JBCs are not skewed towards the anergic B_ND_ subset, suggesting anergy, a mechanism of peripheral tolerance, is not a major force governing JBCs. The B_ND_ compartment is not globally absent in Jo-1 ARS patients; non-JBCs show a similar frequency of B_ND_ cells compared to healthy controls. This suggests specific factors, rather than global host defects, are overriding anergic programming for JBCs.

CD21^lo^ B cells have complex biology. In RA patients, CD21^lo^ B cells have anergic properties [20]. In the context of immunization, CD21^lo^ B cells exhibit properties that suggest they recently exited germinal center reactions and have transcriptional programs that indicate they are poised for plasma cell differentiation [26]. Such activated B cells can drive cognate T cell activation and acquisition of effector function. IgM^+^CD21^lo^ B cells are autoreactive-prone [20, 34] and are increased in the peripheral blood of SLE, RA, and SjS patients [26, 47, 48]. Compared to B cells isolated from healthy individuals, a higher fraction of JBCs from Jo-1 ARS patients had a CD21^lo^ phenotype (Fig. 3). Increased CD21^lo^ B cell frequency correlates with disease severity in SLE and RA [47, 48]. We also found that Jo-1 ARS patients with more severe disease had a trend towards increased frequency of CD21^lo^ cells relative to patients with stable disease, despite the small sample size in these subgroups. Given the contrasting potential functionality of CD21^lo^ B cells (anergy versus plasma cell precursors), we could not infer function from CD21^lo^ phenotype alone in our ex vivo studies. In vitro stimulation drove a lower frequency of JBCs to develop a plasmablast-like phenotype (CD38^hi^CD24^−^) relative to non-JBCs from the same patient, suggesting JBCs may show some degree of functional impairment, but not enough to prevent at least a subset of JBCs from undergoing IgG class switch and plasmablast differentiation.

T cells that proliferate to Jo-1 were found in the peripheral blood of both Jo-1 ARS and healthy controls, suggesting the mere presence of Jo-1-reactive T cells is insufficient to explain disease pathology; rather, Ascherman et al. postulated interaction with professional antigen-presenting cells is likely necessary to invoke pathologic T cell responses [49]. IgM-restricted, autoantigen-binding B cells drive disease in type 1 diabetes-prone mice through their role as antigen-presenting cells, despite their inability to secrete IgG autoantibody [22, 50]. Thus, autoreactive B cell differentiation into antibody-secreting cells may not be required for their involvement in promoting pathologic autoimmune responses. Human IgM^+^ B cells can present antigen to T cells, including IgM^+^ CD21^lo^ B cells isolated from healthy and autoimmune donors [29]. While enrichment of JBCs is increased among IgG^+^ B cells, their predominant detection among IgM^+^ B cells suggests non-class-switched JBCs should be considered when designing new immunomodulatory strategies for Jo-1 ARS, as depletion of plasmablasts or plasma cells would not eliminate antigen presentation and activation of T cells by IgM^+^ (or IgG^+^) JBCs.

In previous studies, total peripheral blood B cell immunophenotyping in patients with Jo-1 ARS demonstrated a decreased frequency of CD19^+^CD27^+^ memory cells (recapitulated here) and increased frequency of naïve B cells in the peripheral blood [42]. Interestingly, B cells infiltrating the muscle of IIM patients exhibited both memory and plasma cell phenotypes [12, 42] and showed evidence of somatic hypermutation, selection, and clonal diversification of their B cell receptors [11, 12]. Our analyses, focused specifically on JBCs, show a higher frequency of JBCs is CD21^lo^ IgM^+^ memory B cells compared to non-JBCs from the same Jo-1 ARS patients.

Our findings highlight the propensity of JBCs to enter CD21^lo^ subsets. CD21^lo^ B cells isolated from rheumatic disease patients upregulate costimulatory molecules and drive CD69 upregulation on T cells, suggesting CD21^lo^ B cells are competent to present antigen to T cells [29]. It is possible JBCs identified within CD21^lo^ subsets are competent to present antigen autoreactive T cells, but this will need to be tested in future work. Detection of JBCs in peripheral blood highlights their potential utility as a biomarker in immunomodulatory clinical trials to allow easy measurement of therapy-induced contraction or phenotypic alteration of autoreactive JBCs.

## Conclusions

Anti-Jo-1 autoantibodies identify the Jo-1 ARS subset of IIM patients. Clinical responses to rituximab implicate B cells in autoimmune pathogenesis. Serologic testing for class-switched Jo-1 autoantibodies identifies Jo-1 ARS patients, but our studies show they do not fully recapitulate the JBC repertoire, as the majority of JBCs have not undergone class switch. These studies define the functional subsets in which JBCs reside to better understand how they expand in Jo-1 ARS patients and highlight their capacity to enter CD21^lo^ and certain memory subsets that are increased in many autoimmune diseases. Our approach also informs the development of future immune therapies; selectively targeting antigen-specific B cells should limit side effects related to broad immune suppression caused by treatments like rituximab. Achieving this goal in Jo-1 ARS requires improved understanding of JBC biology; an optimal approach depends on which functional subset(s) are enriched for this deleterious population of B cells. Additionally, flow cytometry identification of JBCs in Jo-1 ARS patients suggests JBCs could be tracked as biomarkers to assess the efficacy of experimental drugs.

## Supplementary Information

**Additional file 1.**

## Data Availability

The datasets during and/or analyzed during the current study are available from the corresponding author upon reasonable request.

## Abbreviations

ARS: Anti tRNA synthetase syndrome
BCR: B cell receptor
GST: Glutathione S transferase
Jo-1 ARS: Anti-histidyl-tRNA synthetase syndrome
HRP: Horse radish peroxidase
IIM: Idiopathic inflammatory myopathy
Jo-1: Anti-histidyl-tRNA synthetase
JBC: Jo-1-binding B cell
MYSTIC: Myositis and Scleroderma Treatment and Investigative Center
PBMCs: Peripheral blood mononuclear cells
