# Supplementary material for: Jo-1 autoantigen-specific B cells are skewed towards distinct functional B cell subsets in anti-synthetase syndrome patients
Source: Arthritis Res Ther. 2021 Jan 19;23:33. doi: 10.1186/s13075-020-02412-8 (PMC7814460; doi:10.1186/s13075-020-02412-8)
Supplement: Supplementary file 1 — Additional file 1. [file 13075_2020_2412_MOESM1_ESM.pdf]

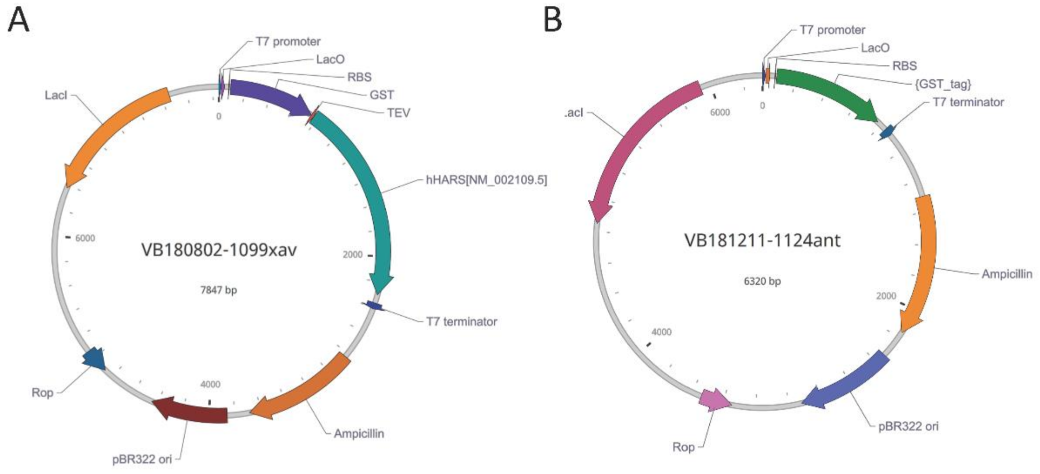

Supplemental Figure 1. Plasmid Constructs for Recombinant Protein Production.
A, To generate the GST-Jo-1 plasmid, the homo sapiens histidyl-tRNA synthetase (hHARS) transcript variant 1 cDNA sequence (NM_002109.5) was purchased from a commercial source (VB180802-1099xav, VectorBuilder). **B,** The GST-tag protein was constructed similarly but only included the GST-tag sequence (VB181211-1124ant, VectorBuilder).

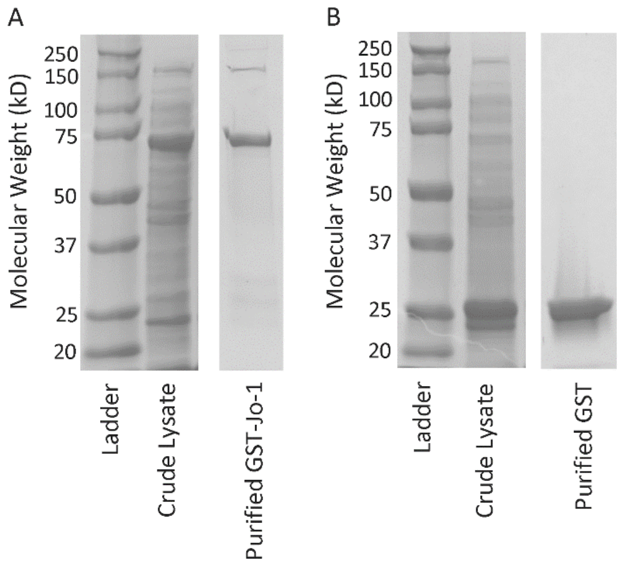

Supplemental Figure 2. Jo-1-GST antigen purification. Proteins were purified using glutathione beads and purity was confirmed by SDS-PAGE gel electrophoresis. Gels were stained with QC Colloidal Commassie (Biorad). Representative gels are shown for purification of **A,** GST-Jo-1 and **B,** GST recombinant proteins.

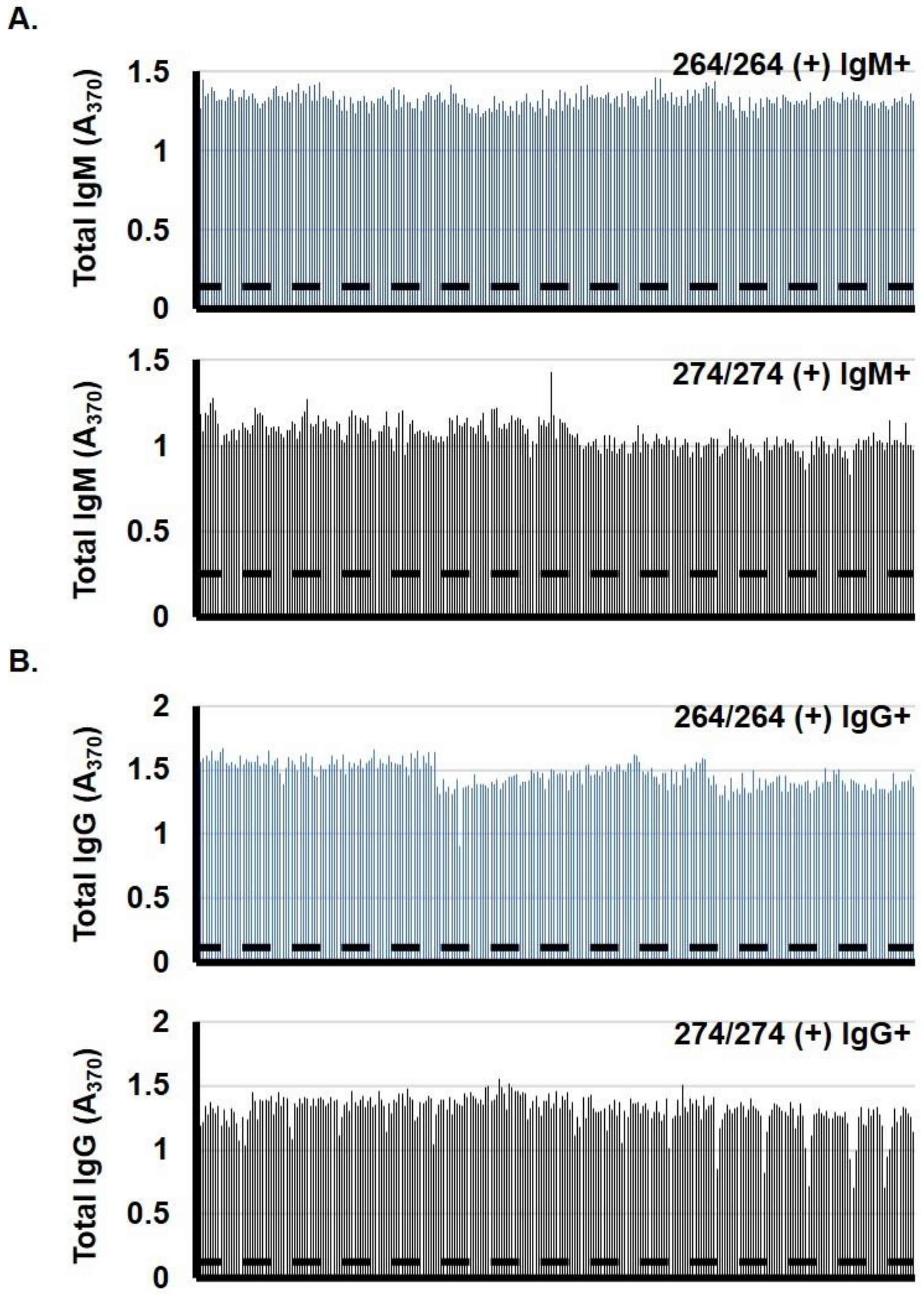

Figure S3. IgM and IgG antibody is detected in stimulated PBMC cultures from subjects with confirmed Jo-1+ disease.

A-B, PBMCs collected from n=5 Jo-1-ARS subjects were separated into individual wells and polyclonally stimulated as in methods to drive B cell differentiation into antibody-secreting cells. Results from two representative patients (one in orange, the other in blue) are shown. **A-B,** Dashed line defines positive wells with a mean fluorescence for background plus 3 standard deviations for plots representing ELISA data from two different patients. The fraction of positive wells is shown secreting **A,** IgM antibody or **B,** IgG antibody.

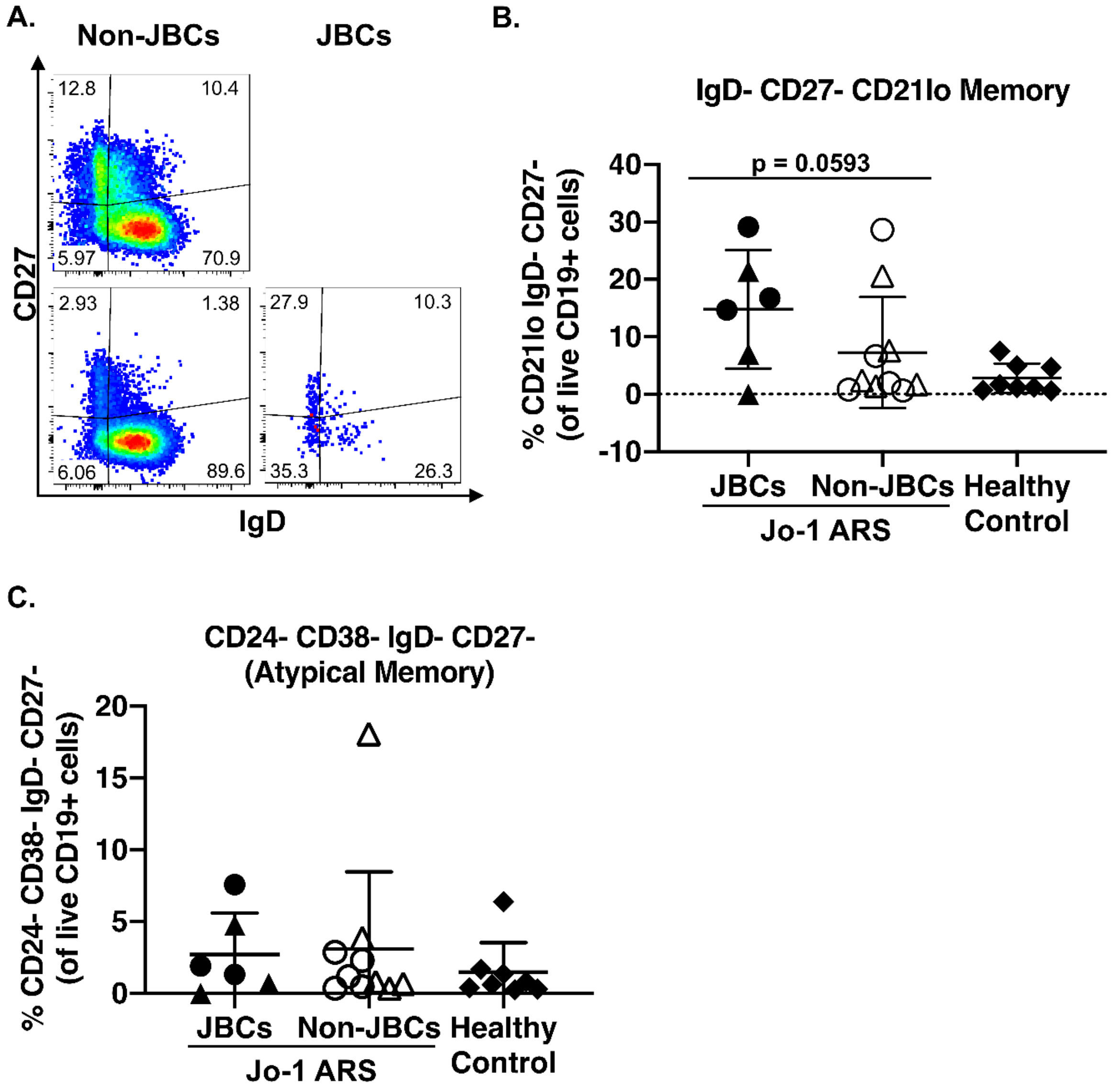

Figure S4. JBCs are not increased among CD21lo CD27- memory b cells or atypical memory.

PBMCs from patients with active healthy controls (top) or Jo-1 ARS (bottom) were stained with antibodies against the indicated antigens and analyzed using flow cytometry and representative plots are shown. **A,** Live, $CD19^{+}IgM^{+}$ Jo-1/GST-binding (JBCs) and were gated on CD27/IgD (Left) expression. **B-C,** Flow cytometry identifies the indicated populations among n=3 stable Jo-1 ARS (triangles), n=3 active Jo-1 ARS (circles), and n=8 healthy controls (diamonds). **B,** The frequency of CD21lo IgD- CD27- memory or **C,** atypical memory (as gated in **Fig. 3A-B)** is shown for JBCs and Non-JBCs isolated from IIM patients as well as healthy control B cells. Each data point represents an individual donor, while the bars represent the mean of each category ± SD. P values were determined using the Mann-Whitney U test, and significant values are indicated on each panel.
